# Great Will Case

**Published:** 1855-07-01

**Authors:** 


### GREAT WILL CASE.

### Dyce Sombre *v.* Troup.

This was a business of proving, in a solemn form of law, the last will and testament, with a codicil thereto, of David Ochterlony Dyce Sombre, Esq., formerly of Sirdhana, in the Upper Provinces of Bengal, in the East Indies, and late of Paris, dated respectively the 25th of June, 1849, and the 13th of August in the same year, and which was originally a business of granting letters of administration, on the suggestion that he had died intestate, promoted by the Hon. Mary Ann Dyce Sombre, widow of the deceased, against Ann May Troup, the sister and next of kin of the deceased, and also against Henry Thoby Prinsep, Esq., one of the executors named in the will.

The documents were propounded in a special allegation, which pleaded that the testator was born at or near Sirdhana, in or about 1808; that his father was of Asiatic extraction, and for some time a colonel in the service and an officer of the household of the Begum Sombre, a princess who exercised rights of sovereignty over a territory in Hindostan; that his mother was the granddaughter of General Sombre, who had been the husband of the Begum Sombre, or who had cohabited with her as such, but who died in her lifetime; that the deceased was, while an infant, adopted by the Begum Sombre, who brought him up in her own palace, and treated him in all respects as her own son; that on the completion of his education, which he received partly under the superintendence of a clergyman of the Church of England, he was admitted by the Begum Sombre to take part in the arrangement of her affairs, and was consulted by her thereon; that, finally, he acquired great power and authority within the territory of the Begum, and continued to exercise the same until her death, which happened in January, 1836; that Mrs. Troup was a natural and lawful sister of the deceased; that Madame Solaroli was also a child of his father, but whether the issue of his mother or of some one else, as frequently asserted by the testator, was unknown to Mr. Prinsep; that Mrs. Troup and Madame Solaroli were also adopted by the Begum, who gave each of them valuable presents on their marriages, which took place on the same day; that the Begum, by her will, dated 1831, after bequeathing several legacies, gave the residue of her real and personal estate to the testator, and requested him to assume the name of Sombre, which he did; that having wound up and settled the affairs of the Begum in the early part of 1838, the deceased came to England; that before leaving the East Indies he placed in the hands of trustees 13,000*l.* to pay the interest to Mrs. Troup for life, and apply the principal at her death for the benefit of her children, and also 10,000*l.* on the same conditions for the benefit of Madame Solaroli; that previously to leaving the East Indies he declared that it was his intention, in accordance with what he knew to have been the desire of the Begum, to bequeath the bulk of the property which he had acquired from her to the East India Company, for the purpose of founding a college for the upper classes of natives, or for some similar purpose, and that he executed a will to that effect which could not now be found; that although he had to some extent received an English education, so as to be in a measure acquainted with the manners and habits of Europeans, yet he was strongly imbued with the feelings peculiar to the natives of oriental countries with respect to the treatment, demeanour, and conduct of women, and retained such feelings, amounting occasionally to fits of uncontrollable passion and jealousy, to the time of his death, and that he was naturally of an irritable and suspicious temperament; that in 1838 he became acquainted with his present widow, a daughter of Viscount St. Vincent, and in the following year made proposals of marriage, which she accepted; and on one occasion the engagement was broken off, but on the 26th of September, 1840, they were married; that he was at the time as well as at all periods prior thereto, of sound mind; that by a settlement in contemplation of the marriage, he transferred to trustees the sum of 133,333*l.* 6*s.* 8*d.* Three per Cent. Consols, to pay the dividends to himself for life, and after his decease to Mrs. Dyce Sombre for life, and in failure of issue of the marriage to his heirs and assigns; that the indenture of settlement was prepared by Mr. Frere, the solicitor who acted for the testator, for Mrs. Dyce Sombre, and for her father; that the indenture was submitted to the testator for his approval, but it was never read over to or by him before its execution; that there was no provision that the benefit taken by her should be accepted in satisfaction of all dower and thirds to which she might become entitled out of his estates as his widow, and that he was not aware of such omission until it came to his knowledge in consequence of certain proceedings in Chancery; that upon being made acquainted therewith he became greatly displeased thereat, and frequently complained that he had not had proper legal advice from the solicitor on the subject; that after the marriage the parties travelled together on the continent, and on their return to England in December, 1840, took up their residence at the Clarendon Hotel, Bond-street; that in January, 1842, Dr. Chambers, who had attended the testator in respect of his bodily health, was consulted by Mrs. Dyce Sombre as to the state of his mind, and, at her suggestion, or with her assent, Dr. Sutherland was called in; that he was frequently visited by those two physicians up to December, 1842; that in February, 1843, Sir James Clark was consulted by her, and he was afterwards assisted by Dr. Conolly and Dr. Monro; that about the 30th of March that year it was finally agreed that he ought to be placed under restraint, and a keeper was appointed to take charge of him; that a commission of lunacy having been procured on the 31st of July following, he was found to have been of unsound mind from the 27th of October, 1842; that the principal portion of the evidence submitted to the jury on the part of Mr. Dyce Sombre, referred to certain insane delusions entertained by him respecting the conduct and behaviour of his wife; that shortly after he had been found of unsound mind, his bodily health began to fail, and his medical attendants advised change of residence; that he finally went to Liverpool, and on the 21st of September escaped from the custody of his keeper and servants, and proceeded alone first to Southampton and then to Paris, where he claimed the protection of the police authorities, which was promised to be afforded him so long as he respected the laws of the country; that Mrs. Dyce Sombre being informed that he was at Paris, Mr. Frere, the solicitor, accompanied by a keeper, went there, and applied to the prefect of police, to cause him to be delivered into his custody; that such application was refused unless he should be able to produce an order to that effect from the Minister of the Interior; that on application being made to him, he directed an inquiry as to the state of mind in which the testator then was; that the testator voluntarily attended such inquiry, and submitted to an examination which lasted upwards of three hours, in the course of which various questions were put to him, founded upon instructions furnished to the prefect by Sir

James Clark and Mr. Frere; that throughout he evinced himself to be of sound mind, memory, and understanding, and the physicians present reported accordingly to the Minister of the Interior, who thereupon declined to interfere with him, or to authorize any restraint being laid on his person or movements; that the funds which he had brought with him from England being soon exhausted, he was obliged to borrow money from persons to whom he was known; that Mr. Frere, previously to leaving Paris, authorized the landlord of the hotel to advance the testator such small sums of money as might be necessary to procure food and clothing and defray any trifling expenses which might be requisite; that in November that year, Mrs. Dyce Sombre authorized Mr. Okey, counsel to the British Embassy at Paris, to examine and settle all the testator's accounts, and provide him with money for his private expenses at the rate of 10*l.* per week; that the testator, being at such time entitled to a clear income of upwards of 18,000*l.* per annum, remonstrated against the insufficiency of such allowance, whereupon Mrs. Dyce Sombre authorized Mr. Okey to honour his draughts for such money as he might require; in consequence thereof, at first, the sum of 16*l.* a week, then 20*l.* per week, and afterwards 28*l.* per week, was paid to the testator over and above his rent and tradesmen's bills, and was continued until May, 1844, when he left Paris for a time; that in January, 1844, he instructed his then solicitor, Mr. Leman, to present a petition to the Lord Chancellor, praying that all the proceedings against him under the commission of lunacy might be superseded, but the petition was dismissed, after hearing counsel, on the 8th of August that year; that in the following December the Lord Chancellor confirmed a report made by Mr. Francis Barlow, one of the Commissioners in Lunacy, and ordered a sum not exceeding 5000*l.* per annum to be expended by the committee of the testator's estate for his maintenance and support, and allowed Mrs. Dyce Sombre 4000*l.* per annum; that, in 1845, after visiting St. Petersburg, Brussels, &c., the testator returned to Paris, where he remained until June, 1846. The allegation then went on to plead in considerable detail various proceedings in Chancery relative to the superseding of the commission, and the allowance to be made to the testator, and averred that in November, 1848, the testator applied to Mr. Prinsep, through whom he had communicated with the Lord Chancellor to recommend him a solicitor to draw up and prepare his will, and he accordingly recommended him to apply to Messrs. Desborough, Young, and Desborough, his own solicitors; that Mr. L. Desborough called upon him at his request, and had a long interview with him, when the testator referred particularly to the proceedings in Chancery arising out of the commission of lunacy, and their effect with regard to his power to make a will; that Mr. Desborough fully discussed with him the sort of evidence which would probably be required to support any will made by him at that time; that the testator at great length stated his wishes in regard to the disposition of his property, and particularly mentioned his desire to make some special provision for the retainers of the Begum, and his wish to found and endow a college for the upper classes of natives in India; that he explained that such his wish was in accordance with that of the Begum, and in compliance with a pledge that he had given; that the testator afterwards committed his instructions to writing, and about the 7th of February, 1849, forwarded the same to Mr. Prinsep, with a note at the end addressed to Mr. Desborough; that on the occasion of Mr. Desborough seeing the testator, he talked and conversed in a rational and sensible manner; that a draught of the intended will was prepared under the advice of counsel, and a fair copy forwarded to the testator at Paris, which he returned with several marginal notes and observations in his own handwriting; that the draught, having been settled in pursuance of the further instructions, was copied fair for execution, and on the 11th of June, and for several days after, Mr. Desborough, jun., conferred with the testator on the subject of his will, and of the various trusts and provisions therein contained; that the same were repeatedly read over to and also by the testator, and were also repeatedly considered by him in the presence of Mr. Desborough, jun., on all which occasions he was of perfectly sound mind; that on the 13th of June he determined on making some addition to his will, in order to appoint a trustee to succeed him with respect to certain trust funds which the Begum had by deeds directed to be applied for religious and charitable purposes, and being unable to find the deeds, he, on the following day, from his own recollection, stated the substance and effect of them to Mr. Desborough, who wrote them down in the margin of the will; that the will, with the additions, having been despatched to London for the purpose of being revised by counsel and recopied, was sent back to Paris; that Mr. Desborough saw the deceased upon it from day to day, and, under his verbal directions, filled in the names of several legatees where blanks had been left, and substituted the senior Roman Catholic priest for the time being at Sirdhana as a trustee in the place of the testator; that after the execution of the will in duplicate both copies were left in the possession of the testator; that before the execution of the will he, at the suggestion of Mr. Desborough, sent for the subscribing witnesses, who were well known to him, and requested them to attend for the purpose of witnessing his will; that, upon one or more of the interviews which Mr. Desborough had with him, the testator spoke of his wife in a manner clearly to show that he was aware of having laboured under unfounded and delusive impressions in respect to her character and conduct, and that his mind was at such time quite free from such impressions, and that he subsequently executed a codicil to his will; that early in the month of December, 1848, being then resident at Mivart's Hotel, Brook-street, he applied to Dr. Paris, the president of the College of Physicians, and to several other physicians of eminence, among whom were some who had given their attention more especially to cases of insanity, to meet in consultation at the hotel, in order that he might be informed of their opinion as to the state of his mind; that he was repeatedly examined, and evinced himself throughout the examinations to be of perfectly sound mind, and fully competent to manage himself and his affairs; that in December, 1848, he returned to Paris, where he thenceforth continued to reside, save for short intervals of a few months at a time, when he travelled for amusement, until he revisited England in 1851, shortly before his death; that while residing at Paris he mixed in society, and was received as a visitor at the houses of his numerous acquaintances in that city, and was visited by them in return, and was always considered and treated by them as a person of sound mind; that in January, 1850, he gave instructions to Mr. Shadwell, his solicitor, to prepare a further petition to the Lord Chancellor to direct a fresh inquiry into the state of his mind, to which his Lordship acceded; but he died before the examination took place; that the several persons, all natives of Hindostan, to whom small annuities in Company's rupees were bequeathed were servants or retainers and dependents of the Begum, or of the testator, and were, during his life, in receipt of monthly pensions granted to them by the testator and paid by his agents in the East Indies; that Anthony Regheliori, a devisee and legatee named in the will, was an officer in the army of the Begum, and highly esteemed by her, and was, after 1838, when the testator left the East Indies, employed by him as his agent to manage his estates and property at Sirdhana and Delhi; that George and John Thomas, also devisees and legatees, were descendants of one George Thomas, who was formerly the head or chief, and exercised rights of sovereignty over a small territory near to that of the Begum, which, on an invasion by a hostile army, he was forced to evacuate, and thereupon took refuge at Sirdhana, where he continued to reside under the protection of the Begum, and he and his family were maintained by her; that the personal estate of the testator in England, France, and the East Indies, which was at his disposal at the time of his decease, and which partly consisted of a balance at his bankers of 7000*l*. and upwards, amounted in value to 500,000*l*. and upwards, independently of claims upon the East India Company to a very large amount.

An allegation was then given in on behalf of the Hon. Mary Ann Dyce Sombre, in which it was pleaded that the parents of the deceased were Roman Catholics; that, after the completion of his education, the society in which he mixed was chiefly composed of the civil and military servants of the East India Company and other Europeans resident at Meerut and Delhi, with their wives and families, with whom, and especially with the ladies, he associated freely, according to English habits and manners, and that one of his most intimate friends was Dr. Drever, then attached to the Begum's household; that the deceased used the European dress, and habitually conformed from his youth to the manners and customs of English society, especially in his treatment of and demeanour to women; that he was naturally of a mild and quiet disposition, gentle and polished in his manners, conduct, demeanour, and conversation; that while in India he was remarkable for his absence of jealousy, and of the peculiar ideas and feelings of the natives of Oriental countries as to women; that he kept a mistress at Sirdhana, and afterwards at Calcutta, and often admitted Englishmen into her apartments while she was therein and was unveiled; that that was a practice wholly at variance with the feelings and habits of natives of Oriental countries with respect to women, and such as no person imbued with those feelings would have adopted; that he manifested no jealousy of her, and never had any fits of uncontrollable passion or jealousy while of sound mind; that Mrs. Troup was a natural and lawful child of the parents of the deceased, and was so treated by the Begum, and also by the testator while of sound mind, and until 1846; that he never before that time expressed any doubt as to the legitimacy of Baroness Solaroli, and spoke and wrote of both his sisters in terms of equal interest and affection; that on his arrival in England, in 1838, he was introduced into the first circles of society, and therein associated freely with ladies and gentlemen, and thenceforth, until 1841, adhered completely to English habits and manners in all respects, and never, until that period, manifested any peculiar jealousy connected with women, save only with respect to his wife; that the marriage settlement was not prepared by Mr. Frere, nor did he act as solicitor for Mrs. Dyce Sombre and her father; that he was recommended to the deceased by Viscount Combermere, who had known the deceased in India; that Mr. Frere only received full instructions from the deceased, and that the draught was submitted and explained to him by Mr. Frere; that he was not possessed of or entitled to any real estate out of which Mrs. Dyce Sombre could claim dower; that Mr. Frere fully explained to him the rights of his wife both as to real and personal estate in case of his dying without children intestate, leaving her surviving, and recommended him to make a will; that he did not, while of sound mind, express any displeasure on account of the settlement, or declare that he had not had proper legal advice from Mr. Frere; that in the spring of 1841 he for the first time became restless, low spirited, fanciful, and suspicious, and at times very much excited, and by his conduct, conversation, and demeanour, evinced, during the remainder of that year, that his mind was in a weak and disordered state; that during such period he frequently and without cause, for the first time, entertained and expressed suspicions of his wife's chastity, both before and since her marriage, and often falsely alleged that she had been abetted in her unchastity by her father and mother; that she had been an opera dancer, and had concealed the fact; that for no cause he applied opprobrious epithets to her, spoke in a loud, violent manner, used threatening gestures to her, and often suddenly, after so doing, fell on his knees before her and asked forgiveness; that at the various periods of the medical examinations referred to in the adverse allegation he laboured under an insane delusion that his wife was habitually unchaste, and that she was frequently guilty of adultery with different gentlemen—among others with her own father; that in 1842, with his concurrence, Dr. Chambers was called in to attend him; and, without the knowledge or privity of Mrs. Dyce Sombre, Dr. Sutherland also saw him; that in March that year, without any cause or reason, on meeting Mr. Alfred Montgomery, with whom he was very slightly acquainted, and who was driving with a lady in a cabriolet, he rushed forward, and in a violent and excited manner endeavoured to stop him; that fears were entertained during such period that he would become insane, but that he slightly recovered during the summer; that in the autumn of 1842, while on a tour in Scotland, he became very unwell, and repeated his suspicions of his wife's criminality with waiters and other persons; that he frequently insisted, notwithstanding denial and explanation, that General Ventura had followed him and his wife to Stafford, and had committed adultery with her at her father's house with his knowledge and sanction; that, while at Inverness, in October, he went into his wife's room where her maid then was alone, to whom he had always before behaved well, seized her, and said she must confess all she knew of her mistress's secrets, or he would murder her; to which she replied, she had nothing to confess; that he thenceforth, and for no other reason, expressed great antipathy to her, and often called her by opprobrious names; that, while so travelling in Scotland, he on various occasions expressed his suspicions that poisons or noxious things had been put into his food, to injure him; that, while at Inverness, he wrote a letter conveying a challenge to General Ventura, on account of the supposed adulteries of his wife with him; that in the following month he sent a challenge to Mr. Montgomery; that in February, 1843, the deceased, being much irritated on account of the East India Company having finally rejected certain claims which he had in right of the Begum upon the Company, sent a memorial to the Queen resigning his claims in her Majesty's favour, and wrote a letter to Sir J. L. Lushington, who had been chairman of the Company, enclosing a copy of it, and desiring him to give him reparation by fighting a duel with him; that he also sent letters to Sir Richard Jenkins, another of the directors, containing a challenge; that in November, 1842, his conduct towards Mrs. Dyce Sombre became so violent as to occasion the greatest apprehensions that he would do her some serious bodily injury; that he would not suffer her to be out of his sight for a minute, for fear, as he said, of her committing some act of adultery in his absence; that at other times he behaved with the greatest affection towards her, and expressed regret for his misconduct; that he talked of being visited by two spirits—one of a benevolent, the other of an opposite character; one desiring him to murder his wife, and the other forbidding it, and telling him he would be happy with her at last; that he was very restless, and often laughed aloud for no reason; that in February, 1843, he called on Dr. Elliotson, to whom he was not known, and desired that the Doctor would make him have more of the company of his (the deceased's) wife, she being also unknown to Dr. Elliotson; that he expressed himself irrationally and violently towards Dr. Elliotson, and insisted on his fighting him; that Dr. Elliotson, having made inquiry respecting him, caused a remonstrance to be made to Mr. Edward Ricketts against the deceased being allowed to go at large: that in March, 1843, it having been determined by medical men, in consequence of his deranged state, to place him under restraint, if Mrs. Dyce Sombre would consent, she reluctantly gave it; that, while under confinement at Hanover-lodge, he continued to manifest the delusion that he was visited by spirits, who conversed with him, and declared that they first appeared to him when he was seven years old, under a pomegranate tree, in the form of the letter T. The allegation then pleaded in great detail a number of insane acts asserted to have been committed by the deceased, and repeated charges made by him of his wife's infidelity both before and after he went to Paris, and averred that in 1847 or 1848 he manifested an insane delusion that the East India Company or some of the directors thereof had tampered with his wife, and had contrived or brought about her infidelity and her incestuous intercourse with her father; that in 1848, being at Rome, he had several audiences of the Pope, and entered into correspondence with Cardinal Franzoni and Dr. Grant on the subject of obtaining a divorce from his wife by reason of her adultery; that, while in London, in 1848, he corresponded and conversed with Cardinal Wiseman on the same subject; that in November that year a meeting took place between the deceased and his wife, in the presence of Mr. Martin and Sir James Clark, at which he suggested that they should come to some understanding about a divorce, and that his conduct and demeanour manifested that he was still labouring under delusion with regard to her unchastity, and also as to the illegitimacy of Madame Solaroli, having first entertained that idea in 1846; that, when at Naples, in 1848, he fancied that he was very ill, that it was occasioned by drugs which had been given to him, and that Baron Solaroli had told him that it would be done at that particular date; that on other occasions he complained of something injurious being put into his food; that at a medical examination, which took place in November, 1848, in London, he evinced that he was of unsound mind; that, having always previously been fastidiously neat and cleanly in his person, and decent in his conduct, in 1846 and 1847 he conducted himself in an indecent and disgusting manner; that, when at Dover and Brighton, in those years, he declared that he would do for his wife; that, while at Brussels, in 1845, he became acquainted with Dr. Mahon, and agreed verbally to pay him 10,000*l.* in case the commission of lunacy should, through his instrumentality and exertions, be superseded, and he should be placed in the uncontrolled possession of his property by the 31st of December ensuing; that, although the commission had not been superseded, so as to entitle him to the 10,000*l.*, yet he claimed a proportion in respect of his services, and in December, 1848, all his claims were referred to the arbitration of Mr. Prinsep and Mr. W. J Richardson, who awarded certain sums, amounting together to 2140*l.*, which were paid; that, being urged by Dr. Bright and Dr. Southey, on an examination before them in July, 1847, to express to Mrs. Dyce Sombre, by letter, the regret which he must feel at having, by his unfounded accusations, so deeply wounded her feelings, he said he would not do so without consulting his lawyers, adding, that if he were to act on the impulse of his own heart, he should never obtain his freedom from the Court of Chancery. After referring to some of the Chancery proceedings alluded to in the adverse allegation, the plea went on to allege that, early in 1849, the deceased caused 2000 copies of a book to be printed, which he had composed and arranged, called "Mr. Dyce Sombre's Refutation of the Charges of Lunacy brought against him," and caused them to be circulated, and that, contrary to the advice of Mr. Prinsep and others, he sent copies of a petition alluding to various passages in the book to peers and members of the House of Commons, stating his readiness to undergo an examination before the British House of Parliament, and praying that such examination should be concluded before the then session was over; that about Easter, 1849, he was introduced to, and saw for the first time, Mr. Forbes Campbell, who had formerly been in the East Indies, when he maintained and persisted, though assured to the contrary, that he had known that gentleman intimately in India; that in August, that year, he declared that a servant was in the room, although he was not; that through the year 1850, and until his death, he continued to labour under the insane delusions as to his wife and the East India Company; that, when in Paris, at various periods from 1845 to 1850, he was accustomed to bring or admit into his rooms, at all hours, common prostitutes; that, while in cabriolets, alone, in 1849 and 1850, at Paris, he talked, swore, and laughed loudly, and often took up prostitutes in the streets to ride with him, who were so alarmed by his conduct and demeanour that they cried out to the driver to stop, and insisted on getting out of such carriage, exclaimed that the deceased was a madman; that in April, 1835, being of sound and disposing mind, he made a will whereby he left everything he possessed to the Begum; that in 1836, after her death, he made another will, the contents of which were not known; that in 1837 he again made a will and disposed of the bulk of his property in favour of Mrs. Troup and her children, and in default of issue in favour of Madame Solaroli and her children, giving about 50,000*l.* to a natural daughter, since deceased, but which will had not been found; that at the time of giving instructions for, and executing the papers now propounded, he was of unsound mind, which he habitually and constantly manifested; that Mr. Prinsep was, in 1845, a candidate for a directorship in the affairs of the East India Company, and was elected a director in 1850; that, while he was a candidate, he often stated and represented that the deceased would benefit India by his will; that in November, 1848, Mr. Prinsep applied to Mr. Lawford, the solicitor of the East India Company, to make a will for the deceased, which he declined to do; that the testator never, at any time before 1843, expressed any respect or gratitude to the East India Company or to the directors thereof, but complained bitterly of their conduct to him, and that he never before that year expressed any intention of leaving any money to the Company or to the directors thereof; that he had a very slight acquaintance with Sir R. Jenkins's daughters, or either of them; that the jewels, trinkets, and ornaments of the person belonging to the deceased, given by the will to his executors, after the determination of the interest therein of Mrs. Dyce Sombre, were of the value of 7000*l.* or thereabouts.

An allegation was also given in on behalf of Baroness Solaroli, which pleaded that she was the lawful sister of the deceased, and co-heiress at law with Mrs. Troup; that the deceased, while in a sane state of mind, knew that his parents were duly married; and that, save in the latter years of his life, when under insane delusion, he, Mrs. Troup, and Baroness Solaroli, constantly owned and acknowledged one another as and for lawful brother and sisters; that the Begum, having adopted the deceased and his sisters from their earliest infancy, treated Mrs. Troup and Baroness Solaroli with maternal affection and as adopted daughters; that they both continued to live in her palace and under her immediate protection until their respective marriages; that their father fell under the displeasure of the Begum, and was dismissed from her service, but for some years afterwards occasionally saw his daughters at the Begum's palace, at Delhi; that on their marriage the deceased assisted at the ceremony, and gave Baroness Solaroli away; that he had kind and affectionate intercourse with them until 1846, and prior to 1838 declared that he intended to leave his property to them, and did so by a will executed that year; that their father died a widower and intestate in 1838, and his property was divided equally between the brother and the two sisters, with the full assent of the deceased; that after he had been found to be a lunatic in 1843, Mr. Francis Barlow, commissioner in lunacy, reported to the Lord Chancellor that Mrs. Troup and Baroness Solaroli were the co-heiresses at law, and only next of kin of the deceased, and, with his wife, were the only persons entitled to his estate, under the statues for the distribution of intestates' estates; that in 1843, when at Paris, he expressed great affection for Baroness Solaroli and her children, and was in the habit of buying toys and presents for the latter; that in 1846 he suddenly took up a fancy that Baroness Solaroli was not his sister; that, under the influence of such insane delusion, he attempted to explain the ground of such notion; that he asserted that Mr. Prinsep had assured him as of his (Mr. Prinsep's) own knowledge that she was illegitimate, but that Mr. Prinsep never had any knowledge of the fact whether she was legitimate or not, save from the assertions of the deceased himself; that the deceased was under the insane delusion that Lord Metcalfe had made an affidavit on the subject, and had left it in the custody of Lord John Russell, and he accordingly several times applied to Lord John Russell for it; that, at a medical examination which took place in 1848, he was examined as to the grounds of his belief that Baroness Solaroli was illegitimate, and his statements evinced that he was then labouring under insane delusions as to his birth; that, while in India, as well before as after the marriage of Baroness Solaroli, he was on terms of great intimacy and friendship with Baron Solaroli, presented him with gifts, and constituted him one of his attorneys for India; that, while at Rome, he solicited the Pope to confer an order of honour on the Baron, to which His Holiness acceded; that in 1846, under the influence of an insane delusion, he maligned and abused the Baron, and printed libels concerning him; that in 1847 he informed the Baron by letter what he had done, and afterwards attacked or menaced him with a stick; that the printer of the libel was fined 200f.; that he again printed the libel, with certain additions and alterations, in his "Refutation;" that the statements contained in it were utterly false and untrue; that Mr. Prinsep and Mr. Desborough, jun., from time to time represented to the deceased that his notions regarding Baroness Solaroli and her husband were considered by physicians and by the Lord Chancellor as delusions, and results and symptoms of his insanity, and, after the date of the will and codicil, they besought him to suppress and conceal them, and warned him that it would be in vain to expect the commission of lunacy would be superseded if he continued to evince them; but he still maintained them, and was under their influence when the will and codicil were executed; that in 1849 and 1850 he continued to circulate the libels regarding the Baron, and in letters to Lord Chancellor Truro exhibited the same delusions; that he was not naturally of an irritable or jealous disposition, but became so after his marriage, and was jealous with regard to women with whom he had cohabited in India, and as to whom, when he cohabited with them, he had shown no jealousy; that after September, 1843, when he escaped to France, he often acted with the most outrageous and revolting indecency, and, among other instances of such conduct, would receive visitors and others in his apartment in a state of nudity, or having only a shirt on, and would exhibit himself out of his apartments in the same state, acting as if unconscious of any impropriety; that he habitually refused to pay for articles which he had purchased, for vehicles which he had hired, and for services ordered by him; in consequence of which, complaints were made to the police authorities, by whom he was treated as a person of disordered intellect.

A further allegation was then brought in on behalf of Mr. Prinsep in support of the papers propounded, in which it was averred that the parents of the testator followed in all respects the usual customs of natives of India, as well in regard to their own conduct as to that of their family and establishment; that it was usual for married ladies of rank, natives of India, to keep slave girls in their harems, and that the mother of the testator had several such slave girls; that, without any violation of the strictest notions of propriety, their husbands were accustomed to cohabit with them as concubines; that, according to the provisions of the Mahomedan law, the children of such concubines were entitled to participate in the distribution of their natural father's property, equally with the children of his lawful wife; that no distinction was made between them; that they were brought up together, and acknowledged each other as brothers and sisters; that Mrs. Troup and Madame Solaroli were taken under the protection of the Begum, and brought up in the usual manner in India; that in 1826, when the Assistant of the East India Company, resident at Delhi, visited them in order to ascertain their wishes in respect to their proposed removal to Sirdhana, he conversed with them from behind a curtain or screen; that the Begum selected and approved their husbands, and they were wholly unacquainted with them previously to their being married to them; that soon after their marriages it was announced by the Begum, or became well known to their husbands, that the testator would inherit the bulk of the property and fortune of the Begum; that frequent discussions arose thereon, and the husbands urged the claims of their respective wives to a portion of the property, and the testator voluntarily promised to make a settlement on them and their children, which he afterwards did; that the testator and the Begum, in his presence, frequently declared that they did not consider that they had any relations who, as such, were entitled to succeed to, or had a right to complain of not sharing the Begum's property; that the testator also declared that if they were content with this promised settlement all well and good, but, if not, they must try their best another way; that after the death of the Begum he was in the habit of declaring that his sisters had no claim on the Begum's property, or upon him as his supposed next of kin, for that his property was given to him by the Begum, and not by his parents; that he resided with the Begum from early childhood, and lived with her down to the period of her death, save only during the time when, as a boy, he resided with the Rev. Mr. Fisher, to whose habits and manners he then temporarily conformed; that such residence produced no permanent change in his habits and feelings, which were essentially those of a native of India, in the condition of life to which he belonged; that, on his attaining a suitable age, the Begum assigned to him, or he took from her harem, with her consent, two or more concubines, and had several children, who all died in their infancy; that they were maintained at the expense of, and in the palace of, the Begum, and were regarded with especial favour by her; that she made them handsome presents, and manifested great grief at their respective deaths; that Mrs. Troup and Madame Solaroli associated freely with the testator's concubines, and took an affectionate interest in the children; that the destruction of human life by the mixture of fatal ingredients with food was a common occurrence among the natives of India; that the upper classes were habitually apprehensive of such a death, and in case of temporary illness habitually attributed it to poison given to kill or injure them; that the testator was at an early age led to believe that his life was in danger, more especially by the Begum, who frequently warned him to take precautions against divers persons, and advised him not to eat anything that might be given him by any person, even by the husbands of his sisters; that the natives of India were habitually superstitious, and had faith in the intervention of spirits and supernatural agency in human affairs; that the testator and the Begum, notwithstanding their profession of Christianity, were, and continued at all times to be, impressed with such belief; that the testator's father, on being dismissed from the service of the Begum in 1826, brought forward certain pecuniary claims, amounting to a considerable sum, against her, but which were not admitted during her life and remained unsettled at her death, when they were preferred against the testator as the successor to her property and fortune; that previously to his leaving Calcutta, on his voyage to England, his father commenced proceedings against him, but which were abandoned upon an engagement made with his sanction to pay a certain sum of money; that that sum formed a considerable part of the personal estate to the father; that the testator received no distributive share of his father's personal estate, but as the third part was collected and got in, it was set off against the sum of money due and owing from the testator; that shortly after his marriage the testator admitted that he was jealous of his wife, especially in August, 1842, in the presence of General Ventura, to whom he declared that he bitterly regretted the usages of English society, which compelled him to allow his wife to go into company, where she was exposed to the attentions of other men; that at the same time he became suspicious and distrustful of those men whom his wife met in society, and even of General Ventura, by reason of his having, when in conversation with the testator, expressed himself in strong terms of admiration of her; that such distrust and suspicion were admitted by his wife, after consulting her friends, to arise from his ignorance of the manners and habits of society in Europe; that previously to his marriage he challenged his wife's father in consequence, as he averred at the time, of her having broken off her engagement to marry him; that in 1843, shortly after Baron Solaroli and his wife arrived in England from India, but before the execution of the commission of lunacy, the baron sent a letter to the testator, wherein he offered his services to him on the occasion of such commission being issued, but which offer was rejected by the testator; that on the testator escaping to Paris, Baron Solaroli went to him from Boulogne, where he was resident, and was present at the examination before the prefect of police, previously to which he sent a letter to the French Minister of the Interior, wherein he solicited the protection of the French laws on behalf of the testator against the attempts of his wife and her agents to regain possession of his person, and stated, among other things, that the testator's conduct protested loudly against the pretended madness which had been imputed to him, and which could have no object but to deprive him of his fortune; that when application was made to the Court of Chancery for the appointment of a committee of the person and estate of the testator, in 1843, Mr. Troup, being fully informed by Baron Solaroli of the then state of the testator's mind, wrote several letters to the testator, offered his services in communicating his views to his lawyers, suggested that the testator had not been fairly dealt with, and referring to the verdict of the jury, expressed his sincere regret that he should be so fettered; that when *ad interim* committees of the person and estate of the testator were appointed, Mrs. Dyce Sombre sent a letter to Mr. Okey, then at Paris, and therewith transmitted copies of several affidavits in respect of his property, and stated that they were sent for the perusal of the testator; that if any part of a schedule she forwarded was wrong as to the Indian property, she requested Mr. Okey to get the testator to explain it, and let her know, that she might have it rectified; that Baron Solaroli, notwithstanding he had exerted himself in 1843 on behalf of the testator, nevertheless co-operated with Mrs. Dyce Sombre and Mr. Troup in opposing him in all the proceedings subsequently taken to supersede the commission of lunacy, in consequence of which the testator conceived a great dislike and antipathy to him, which he constantly expressed; that entries of the baptism of the testator and Mrs. Troup were duly made in the register book kept for the church of the Roman Catholic Mission of Sirdhana, but no entry could be found of the baptism of Madame Solaroli, and the deceased frequently alluded to that as a ground for believing that she was illegitimate; that before he entered into the engagement with Dr. Mahon, he voluntarily submitted himself to the examination of several medical men, and evinced himself to be of perfect sound mind, memory, and understanding; that while at Brussels, in 1845, he mixed in society, and was considered by friends, acquaintances, and medical men to be of sound mind, and competent to manage himself and his affairs; that Mr. Prinsep, who was at the time Secretary of the Government of India, and in attendance on the Governor-General of India, first became acquainted with the testator in 1831, when the Begum and her troops, then in command of the deceased, joined the camp of the Governor-General; that upon his arrival at Calcutta, in 1837, where Mr. Prinsep, who then held the office of Secretary to the Government of India in the General and Financial Department, was at that time residing, the testator renewed his acquaintance with him, and consulted him as to the investment of his property; that at a late hour one night, when he could not procure the necessary bail for his release, he was arrested at the suit of his father, and the fact becoming known to Mr. Prinsep, he took measures to enable him to procure his release, for which the deceased expressed his gratitude; that, with few exceptions, he had no further intercourse with him until 1844, when he accidentally met him in London; that knowing the testator to be a proprietor of East Indian Stock, he solicited him for his vote and interest for the office of Director; that he informed Mr. Prinsep of the Chancery proceedings, and occasionally corresponded with him in respect of his affairs, but did not interfere therein, except to apply to the Lord Chancellor to allow him a better income, which was granted; that in the latter end of 1845, or the beginning of 1846, Mr. Mahon, who was then unacquainted with Mr. Prinsep, called upon him in London, and stated the terms of his engagement with the testator, and of which he had been apprised by a letter from the testator himself; that Mr. Mahon informed Mr. Prinsep of the intention of the testator to renew his application to supersede the commission of lunacy, and showed him the opinions of medical men, and other statements and evidence on which his expectation of success was founded; that Mr. Prinsep being thereby led to think more favourably of the case of the testator than he had previously done, he visited him in 1844, both at Dover and Paris, and saw him on several occasions, on each of which his conduct and conversation were rational and sensible; that about January in that year, he expressed a desire to make his will, and made particular inquiries of Mr. Prinsep as to who would succeed to his property in case he left no will; that Mr. Prinsep, in reply thereto, informed him that his wife and sisters would divide it equally between them, but, as regarded his wife, provided her claims were not barred by her marriage settlement, and as regarded his sisters, provided they were born of the same mother, and that his father's marriage with his mother could be proved; that he requested Mr. Prinsep to make inquiries of Lord Metcalfe, who, as he stated, knew everything respecting their birth and parentage; that Mr. Prinsep had two interviews with his Lordship, who said he believed that the testator and Mrs. Troup were children who were introduced to him at the Begum's house, at Delhi, but he could give no information about any younger child; that Mr. Prinsep informed the testator of the purport and effect of the statement of Lord Metcalfe, who in reply expressed his surprise at the reserve of his Lordship, for that he must have known the circumstances relating to the birth of Madame Solaroli, and that she was the daughter of his father by a slave girl; that in consequence of the death of Lord Metcalfe, Mr. Prinsep was unable to consult him again; that after the first mention to Mr. Prinsep of his wish and intention to make a will, he repeated it on several subsequent occasions, both verbally and by letter, and requested Mr. Prinsep to ask the solicitor to the East India Company to go to him at Paris for that purpose; that Mr. Prinsep accordingly applied to Mr. Lawford, who declined to make such will, or to advise the testator in reference thereto, by reason of the testator having some unsettled claims upon the East India Company which it might be his duty, upon the part of the Company, to resist; that the testator, when in London, in November, 1848, again adverting to the subject, asked Mr. Prinsep to procure from his own solicitors a form of a will which he might fill up, but, on applying to Messrs. Desborough and Co., he was informed that no such form could be recommended, and upon further communication with him, he requested Mr. Prinsep to desire one of the firm to call upon and converse with him on the subject; that the testator never spoke to or consulted Mr. Prinsep as to the contents of his intended will, and he was ignorant of the purport of it until after his death, save that he informed him after its execution that he was appointed one of the executors; that in or previous to 1848, Mr. Mahon, in consideration of services which he had rendered to the testator, in procuring him the enjoyment of his entire surplus income, claimed to be reimbursed his expenses, and to be compensated for his losses and trouble; that the testator proposed to refer the claim to arbitration, and with a full knowledge of the opinion of Mr. Prinsep in respect of the justice of the claim, and a concurrence in that opinion, urged Mr. Prinsep to accept the office of arbitrator, which he ultimately consented to do, and Mr. Richardson was appointed by Mr. Mahon to act on his behalf; that throughout the proceedings and inquiry before the arbitrators, the deceased evinced himself to be of sound mind; that in the spring of 1851, Dr. Winslow, who was then staying at an hotel at Paris, was visited by the testator, and requested to examine him in case there should be any dispute about his will; that Dr. Winslow had long and repeated interviews with him on several successive days, during about three weeks; that the testator discussed with him the various proceedings to supersede the commission of lunacy and the result, and produced to him various affidavits and other documents connected therewith, and freely and unreservedly conversed with him thereon, and especially in respect to his wife, and Madame Solaroli and her husband, and as to his alleged delusions in regard to them; that the testator throughout such interviews evinced himself to be of perfect sound mind; that the testator, when at Paris and elsewhere on the continent of Europe, was watched by persons employed for that purpose, by or on behalf of his wife, which were used in opposing the petitions presented by him to the Court of Chancery, and that the testator was well aware of it; that Mr. Charles Shadwell, his solicitor, in reference to several affidavits filed in opposition to the last petition, which came on for hearing in May, 1851, informed him that they had been treated by the Court with the contempt they deserved, and that the petition had been opposed in every possible way; that on his arrival in London in 1851, he complained of such, the conduct and proceedings of his opponents, and especially of his wife; and at an interview which he had with Mr. Prinsep, produced and commented in severe terms upon the contents of a note which he had just received from her, in which she solicited an interview with him; that he had associated with common prostitutes in India, and that occasionally, while resident in the Upper Provinces, he had appeared in his dwelling-house and in places of public resort in a state of nudity, or without any article of clothing except a "lungotee" fastened round his loins and hips; that from an early period of life he was in the habit of making use of oaths and profane and angry expressions without cause or provocation; that though lavish in his expenditure, or in his presents to the women with whom he cohabited, and also upon his amusements, yet he was at all times careful in the investigation of pecuniary demands upon him; and that in regard to his habits and conduct there was no change at any time prior to his decease.

